# Clinical Laboratory Perspective on *Streptococcus halichoeri*, an Unusual Nonhemolytic, Lancefield Group B Streptococcus Causing Human Infections

**DOI:** 10.3201/eid2705.203428

**Published:** 2021-05

**Authors:** Salika M. Shakir, Rahul Gill, Jonathan Salberg, E. Susan Slechta, Mark Feldman, Thomas Fritsche, Jill Clarridge, Susan E. Sharp, Mark A. Fisher

**Affiliations:** University of Utah/ARUP Laboratories, Salt Lake City, Utah, USA (S.M. Shakir, M.A. Fisher);; Texas Health Presbyterian Hospital of Dallas, Dallas, Texas, USA (R. Gill, M. Feldman);; Kaiser Permanente Regional Microbiology and Molecular Infectious Diseases Laboratories, Portland, Oregon, USA (J. Salberg, S.E. Sharp);; ARUP Institute for Clinical and Experimental Pathology, Salt Lake City (E.S. Slechta);; Marshfield Clinic Health System, Marshfield, Wisconsin, USA (T. Fritsche);; University of Washington, Seattle, Washington, USA (J. Clarridge);; Copan Diagnostics, Murrieta, California, USA (S.E. Sharp)

**Keywords:** antimicrobial resistance, bacteria, underlying health conditions, grey seals, streptococci, Streptococcus halichoeri, zoonoses

## Abstract

*Streptococcus halichoeri* is a relatively newly identified species of pyogenic streptococci that causes zoonotic infection in humans. *S. halichoeri* was first described in 2004 as indigenous to seals, and only 8 reports of human *S. halichoeri* infection have been published. *S. halichoeri* grows as small, white, nonhemolytic colonies and may be strongly catalase-positive on routine blood agar media, which can lead to isolates being misidentified as coagulase-negative staphylococci. *S. halichoeri* tests positive for Lancefield group B antigen, like *S. agalactiae*, but can be identified with matrix-assisted laser desorption/ionization time of flight mass spectrometry or partial 16S rRNA sequencing. We describe 3 cases of *S. halichoeri* bone and joint infections in patients in the United States with underlying health conditions. In addition, we examine the microbiologic characteristics of *S. halichoeri* and discuss the importance of fully identifying this organism that might otherwise be disregarded as a skin commensal.

*Streptococcus halichoeri* is a recently identified member of the genus *Streptococcus* capable of causing pyogenic human infections. It was first isolated from gray seals (*Halichoerus grypus*) and formally described in 2004 (*1*). In 2013, it was reported as the causative organism of empyema in a patient with diabetes (*2*). *S. halichoeri* differs from other pyogenic streptococci in that it is nonhemolytic and may exhibit strong catalase activity when grown in blood-containing media such as chocolate and sheep blood agar (SBA). These phenotypic characteristics, along with its colony and Gram-stain morphology, may lead to its misidentification as a coagulase-negative *Staphylococcus*. In addition, *S. halichoeri* is positive by Lancefield group B–typing assays but can be distinguished from *S. agalactiae* by its Gamma hemolysis and negative hippurate hydrolysis results (*2*). A 2016 study described distinct phenotypic and genetic differences between 6 human clinical isolates and the type strain from a gray seal. The authors proposed distinct subspecies for human (*S. halichoeri* subspecies *hominis*) and grey seal isolates (*S. halichoeri* subsp. *halichoeri*) (*3*). Our study examined recent clinical experience with *S. halichoeri* as a cause of bone and joint infections in patients in the United States with underlying health conditions. We describe phenotypic, genetic, and antimicrobial susceptibility characteristics of *S. halichoeri* recovered from clinical specimens.

## Materials and Methods

### Bacterial Isolates

We included in this study 45 *S. halichoeri* isolates from 39 patients identified during 2010–2018 at ARUP Laboratories, a national clinical reference laboratory. Sixteen isolates were identified by DNA sequencing and 39 by matrix-assisted laser desorption/ionization time of flight (MALDI-TOF) mass spectrometry. We selected 5 isolates available in the strain repository for further characterization with traditional biochemical tests including pyrrolidonyl arylamidase (PYR), hippurate hydrolysis, and bile-esculin growth/hydrolysis (*4*). We determined Lancefield type using the Hardy StrepPro Grouping kit (https://hardydiagnostics.com). This study was approved by the University of Utah Institutional Review Board (no. 24431).

### Antimicrobial Susceptibility Testing

Antimicrobial susceptibility testing (AST) results were available for 22 clinical isolates. We tested the isolates using Sensititer custom broth microdilution panels (https://www.thermofisher.com) in cation-adjusted Mueller-Hinton broth supplemented with 5% lysed horse blood. We determined MIC values for penicillin, ceftriaxone, daptomycin, vancomycin, clindamycin, erythromycin, levofloxacin, meropenem, doxycycline, and quinupristin/dalfopristin and interpreted results according to Clinical and Laboratory Standards Institute (CLSI; https://clsi.org) guidelines for viridans group *Streptococcus* species (CLSI M100 2019A). We performed quality control using *S. pneumoniae* ATCC 49619 according to CLSI guidelines (*5*).

### MALDI-TOF Mass Spectrometry

We performed MALDI-TOF mass spectrometry as described elsewhere (*6*). Isolated colonies from Hardy Columbia sheep blood agar incubated at 35°C and 5% CO_2_ were spread only on a polished steel target and overlaid with 1 µL of matrix (α-cyano-4-hydroxycinnamic acid in 50% acetonitrile, 47.5 % water, and 2.5% trifluoroacetic acid) and air dried. We collected mass spectra as described elsewhere (*6*); each spectrum was a sum of 240 shots collected in increments of 40. We analyzed spectra using the Bruker Biotyper commercial database (https://www.bruker.com) and used scores of ≥1.9 for identification to the species level (*6*). Custom mass spectral profiles (main spectra, or MSPs) were created according to manufacturer’s recommendations. In brief, we prepared extracts from 5–10 mg of cells using the standard formic acid–acetonitrile method; 10 replicate 1 µL aliquots were air dried on a polished steel target, then overlaid with 1 µL α-cyano-4-hydroxycinnamic acid and air dried. We collected spectra from each spot in triplicate and reviewed them in Bruker FlexAnalysis, then processed 24 spectra for MSP generation in Bruker CompassExplorer using default settings.

### 16S rRNA Gene Sequencing

We extracted bacterial DNA from 3 McFarland standard suspensions with the PerkinElmer Chemagic MSM-automated extraction platform (https://chemagen.com), according to the manufacturer’s instructions. Initially, we generated the first 500 bp of the 16S rRNA gene using 5F-T and 534R-T primers and subsequently generated nearly full-length 16S sequences with 5F, 357F, 534R, 806F, 1053R, and 1492R primers (*7*). We analyzed sequences using Pathogenomix RipSeq (https://www.pathogenomix.com) and BLAST (https://blast.ncbi.nlm.nih.gov). We performed phylogenetic analyses and generated neighbor-joining trees on partial and full-length sequences using MEGA version X software (https://www.megasoftware.net) (*8*).

### *S. halichoeri* Clinical Context

We describe 3 representative clinical cases of *S. halichoeri* bone and joint infection to highlight the clinical disease progression and microbiological characteristics of this unusual organism. The first case-patient was a 43-year-old man with poorly controlled type 2 diabetes mellitus, hypertension, and stage 3 chronic kidney disease who sought treatment for a 7-week history of low back pain radiating to his legs. Spinal magnetic resonance imaging (MRI) revealed diffuse edema of the fourth lumbar vertebral body with a possible fracture. Results from fluoroscopy-guided vertebral biopsy were unremarkable. He was discharged with plans for a follow-up MRI in 1 month but was readmitted 2 weeks later with worsening low back pain. Leukocyte count was within reference range; erythrocyte sedimentation rate (110 mm/h; reference <20 mm/h) and C-reactive protein level (CRP; 5.79 mg/dL; reference <0.5 mg/dL) were elevated. MRI results suggested L3 and L4 vertebral osteomyelitis and diskitis. Fluoroscopy-guided L4 vertebral biopsy had negative stains and cultures for bacteria, mycobacteria, and fungi. Additional biopsies from L4 bone, L3–4 disk, and both posterior paraspinal areas were negative by Gram stain, but cultures from all 4 yielded gram-positive cocci in clusters that grew as tiny, whitish-gray, nonhemolytic colonies on SBA. The isolate was initially reported as coagulase-negative *Staphylococci* on the basis of phenotypic testing but later identified as *S. halichoeri* by MALDI-TOF mass spectrometry (Bruker Biotyper 5627 database) at ARUP Laboratories. The isolate (229 in this study) was catalase-positive when grown in SBA, PYR-positive, weakly positive for Lancefield B antigen, and hippurate hydrolysis negative. Gram stain from SBA showed cocci, mostly in clusters with a few small chains, but revealed substantial chaining when grown in broth, and catalase testing from Mueller-Hinton agar was negative; both findings were consistent with those for most members of the genus *Streptococcus* (Table 1). The isolate was sensitive to all antimicrobials tested, including penicillin, vancomycin, levofloxacin, and linezolid. Because of a penicillin allergy, the patient was treated with renally adjusted vancomycin (1,500 mg/18 h), but he continued to have back pain, and new weakness developed in his legs after 5 weeks of therapy. Subsequent MRI revealed disease progression with diffuse marrow edema and end-plate disruption of the L3–4 vertebrae along with phlegmon formation and myositis of both psoas muscles, prompting addition of meropenem to his treatment. Needle biopsy of L4 vertebrae revealed reactive bone with degenerative changes and chronic inflammation, but routine bacterial cultures were negative. The patient’s symptoms improved after 7 additional weeks of intravenous vancomycin and meropenem. This treatment was followed by 6 weeks of oral minocycline and cefuroxime, during which time his back pain was alleviated and inflammatory markers normalized.

**Table 1:** Phenotypic characteristics of 5 *Streptococcus halichoeri* human clinical isolates, United States*

Isolate	Hemolysis	Catalase-SBA	Catalase-MHA	PYR	Esculin	Bile-esculin	Hippurate	Lancefield B antigen
018	Negative	Positive	Negative	Positive	Positive	Positive	Negative	Weak positive
116	Negative	Negative	Negative	Positive	Positive	Positive	Negative	Weak positive
076	Negative	Weak positive	Negative	Positive	Positive	Positive	Negative	Weak positive
229†	Negative	Positive	Negative	Positive	Positive	Positive	Negative	Weak positive
853‡	Negative	Positive	Negative	Positive	Positive	Positive	Negative	Weak positive

*MHA, Mueller-Hinton agar; PYR, pyrrolidonyl arylamidase; SBA, sheep blood agar. †Isolate from patient described in case 1. ‡Isolate from patient described in case 2.

The second case-patient was a 68-year-old man admitted for planned removal of his infected left knee arthroplasty and left knee fusion. The patient had a history of hypertension and giant cell tumor of the left knee 3 years earlier; at that time, left total knee arthroplasty, curettage, and polymethylmethacrylate packing of the left tibia were performed without complications. Three months before his admission for the removal, the patient noted purulent draining lesions around the surgical site for which he was prescribed amoxicillin/clavulanate. Initial radiographs showed no evidence of tumor recurrence and stable-appearing changes of the left proximal tibia and left total knee arthroplasty. Chronic prosthetic joint infection was suspected, as well as left knee arthrofibrosis. Because of a rash attributed to amoxicillin/clavulanate, the patient was switched to trimethoprim/sulfamethoxazole, which was stopped ≈2 weeks before the planned second left knee arthroplasty. During the prosthetic joint removal procedure, knee fluid and resected tissue sent for culture which grew 3 organisms: methicillin-susceptible *Staphylococcus aureus* and *S. epidermidis,* both identified by conventional phenotypic methods, and a third organism from thioglycolate broth that stained as gram-positive cocci in chains. This organism, which grew as white, nonhemolytic colonies on SBA and chocolate agar at 48 hours, was positive for catalase, PYR, leucine aminopeptidase, and bile-esculin and negative for growth in media with 6.5% salt. The organism (isolate 853 in this study) was sent to ARUP laboratories, where it was identified as *S. halichoeri* by 16 S rRNA sequencing. Eight separate cultures of the knee tissue grew *S. halichoeri,* methicillin-susceptible *S. aureus,* and *Finegoldia magna.* AST of *S. halichoeri* by broth microdilution revealed pansusceptibility to drugs tested for viridians group streptococci. The patient received treatment for the polymicrobial infection with intravenous ceftriaxone and oral rifampin for 6 weeks.

The third case-patient was a 68-year-old man with a pertinent history of poorly controlled type 2 diabetes mellitus with peripheral neuropathy and suspected arterial insufficiency, seeking treatment for a severe diabetic foot infection and altered mental status. The patient fulfilled 3 of 4 criteria for systemic inflammatory response syndrome: fever of 101.2°F, tachycardia (112 bpm), and leukocytosis (leukocytes 18 k/μL). He had elevated CRP (77 mg/L), erythrocyte sedimentation rate with reference range (21 mm/h), and elevated blood glucose (248 mg/dL; reference fasting glucose <100 mg/dL). Aside from confusion, the patient had edema, erythema, and pain of the right second toe. No previous history of chronic wound or drainage in this area was noted and the wound was provisionally attributed to a nail grinder. Radiographs showed no signs of osteomyelitis. The patient was started on broad-spectrum intravenous therapy with vancomycin and ampicillin/sulbactam, and he showed marked improvement in the edema and erythema of the toe. Blood cultures collected at the time of examination were negative, however cultures of a right second-toe abscess were positive for gram-positive cocci in chains. The organism grew as small, white, nonhemolytic colonies that were weakly positive for slide coagulase but tube coagulase negative. Catalase-positivity was observed when the organism grew on SBA and chocolate agar but not when grown on tryptic soy agar. The organism was typed as Lancefield group B by latex agglutination and was initially identified by the VITEK 2 system (bioMéerieux, https://www.biomerieux.com) as *Streptococcus suis*. Because of the discrepancy between the Lancefield typing and VITEK 2 results, we performed 16S rRNA sequencing, which identified the organism as *S. halichoeri*. Unfortunately, the isolate was not available for further testing in this study. The patient was transitioned to a 14-day course of oral trimethoprim/sulfamethoxazole and amoxicillin/clavulanate, and by 4 months after debridement, the patient’s wound had healed.

### *S. halichoeri* Infection Sites

We analyzed 45 *S. halichoeri* isolates from 39 patients identified during 2010–2018 at ARUP Laboratories. Most (n = 28, 71%) isolates were from male patients; 18 isolates (40%) were from wound infections, 9 (20%) from blood specimens, 7 (16%) from tissue, and 4 (9%) each from normally sterile body fluids (1 peritoneal fluid, 2 knee fluid, and 1 unspecified) and urine (9%). Of the 9 patients with positive blood cultures, 3 were also positive for the organism in urine, foot wound, or knee fluid cultures.

### Microbiologic Characteristics of *S. halichoeri* Isolates

We performed a retrospective review of the microbiology results from the laboratory information system on 45 isolates of *S. halichoeri*. The laboratory performed Gram stains on all isolates, which were reported as gram-positive cocci in chains or clusters. All but 1 isolate failed to exhibit hemolysis on SBA plates. Most isolates (n = 29, 64%) were identified by MALDI-TOF mass spectrometry with the remainder (n = 18, 40%) by partial 16s rRNA gene sequencing. We selected 5 isolates available in our strain repository (including isolates from case-patients 1 and 2) for further characterization with biochemical and molecular methods (Table 1). Four of these isolates were catalase-positive when grown on SBA, but all 5 isolates were catalase-negative when grown on Mueller-Hinton agar lacking blood supplementation. All 5 isolates were PYR-positive, bile-esculin and esculin positive, hippurate-negative, and weakly reactive with Lancefield group B antiserum.

Antimicrobial susceptibility testing results were available for 22 clinical isolates (Table 2). All isolates were susceptible to ceftriaxone, daptomycin, levofloxacin, linezolid, meropenem, penicillin, and vancomycin. Six isolates (28%) were resistant to clindamycin and nonsusceptible to erythromycin (5 resistant, 1 intermediate).

**Table 2:** Antimicrobial susceptibility profiles for 22 human clinical isolates of *Streptococcus halichoeri*, United States*

Antimicrobial agent	MIC values, µg/mL			
	% Susceptible	Range	MIC_50_	MIC_90_
CRO	100	≤0.06–0.25	0.12	0.25
CLI	77.3	≤0.03 to ≥4	0.03	≥4
DAP	100	≤0.12–0.5	0.25	0.25
DOX†	NA‡	≤0.25–16	0.25	0.5
ERY	72.7	≤0.12 to ≥8	0.12	2
LVX	100	≤0.25–2	1	1
LZD	100	0.5–1	1	1
MEM	100	≤0.06–0.25	0.06	0.06
PEN	100	≤0.03–0.06	0.03	0.03
Q/D‡	94.7	≤0.25 to ≥4	0.25	0.25
VAN	100	≤0.5–1	0.5	0.5

*CLI, clindamycin; CRO, ceftriaxone; DAP, daptomycin; DOX, doxycycline; ERY, erythromycin; LVX, levofloxacin; LZD, linezolid; MEM, meropenem; MIC_50_, MIC value at which ≥50% of the isolates in a test population are inhibited; MIC_90_, MIC value at which ≥90% of the isolates in a test population are inhibited; NA, not available; PEN, penicillin; Q/D, quinupristin/dalfopristin; VAN, vancomycin. †Clinical and Laboratory Standards Institute interpretive breakpoints for doxycycline were not available for viridans group *Streptococcus* spp. If breakpoints for *S. pneumoniae* were applied, 82% would be susceptible (*5*). ‡n = 19 isolates.

### 16s rRNA Gene Sequence Analysis of *S. halichoeri* Isolates

**Figure 1:**
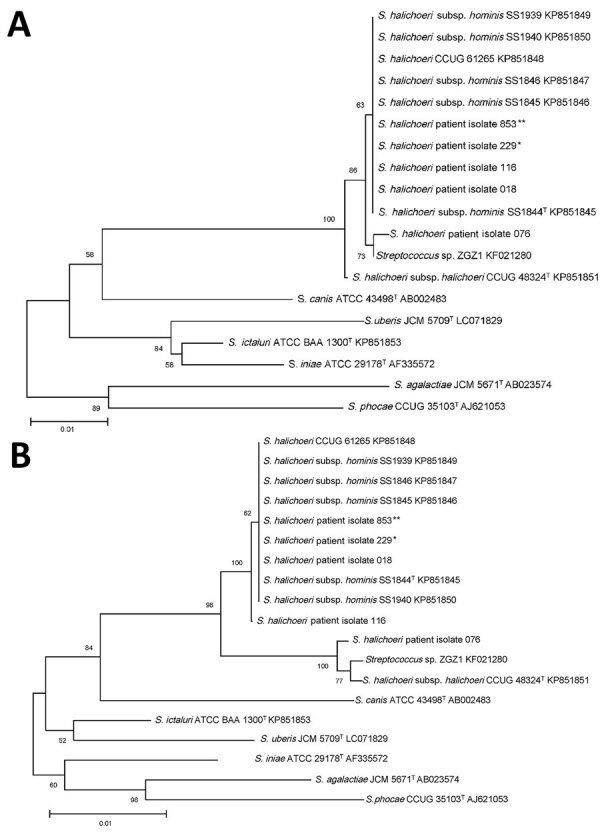
Phylogenetic trees based on 16S sequences of clinical and type strains of *Streptococcus halichoeri* and related taxa from study of human infections caused by unusual strains of *S. halichoeri*, United States. A) Partial sequences (496 nt); B) full-length sequences (1,434 nt). We generated alignments using ClustalW (http://www.clustal.org), trimmed them to the length of the shortest sequence, and computed neighbor-joining trees with bootstrap analysis with 1,000 replicates using MEGA X (https://www.megasoftware.net). Isolates from case-patients are represented with asterisks (*patient 1; **patient 2). ^T^ indicates type strains.

We performed partial and near full-length 16S ribosomal RNA gene sequencing on the 5 selected clinical isolates. Partial 16S sequences of all isolates shared 97% identity with *Streptococcus canis* (ATCC 43496) and 96% similarity to *Streptococcus ictaluri* (ATCC BAA-1300) and *Streptococcus iniae* (ATCC 29178). Four isolates (018, 116, 076, and 229) were 99% identical to the partial 16S rRNA sequence of *S. halichoeri* strain M512/02/1 (type strain, CCUG 48324), however, isolate 853 was only 98% identical. With near full-length 16S rRNA sequence analysis, we observed the subspecies distinction proposed in a 2016 study (*3*). Isolates 018, 116 (GenBank accession no. MT771643), 229, and 853 were 98.5%–98.6% identical to the *S. halichoeri* subsp. *halichoeri*–type strain (GenBank accession no. KP851851) but were more closely related to clinical isolates characterized as *S. halichoeri* subsp. *hominis* (*3*). Phylogenetic analysis showed the tight clustering (99.9%–100% identity) of isolates 018, 116, 229, and 853 with the proposed *S. halichoeri* subsp. *hominis*–type strain (KP851845) and the 5 isolates from patients in the 2016 study (*3*) (Figure 1). In contrast, isolate 076 was more closely related to the *S. halichoeri* subsp. *halichoeri*–type strain (CCUG 48324, 99.7% sequence identity) and a *S. halichoeri* isolate from a badger with pyogranulomatous pleuropneumonia (GenBank accession no. KF021280, 99.9%) (*9*). Isolate 076 (GenBank accession no. MT771642) had 19–20 base substitutions distinct from the previously described *S. halichoeri* subsp. *hominis* isolates and isolates 018, 116, 229, and 853 (98.6%–98.7% identity), suggesting it is more closely related to animal than human strains.

### Mass Spectra of *S. halichoeri* Isolates

**Figure 2:**
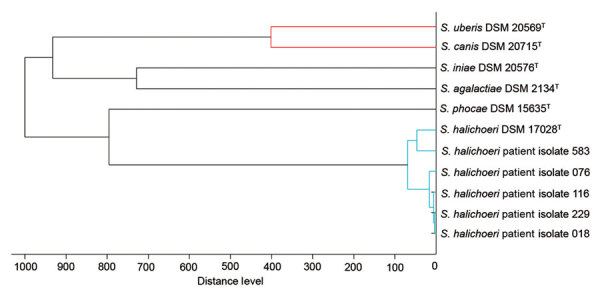
Matrix-assisted laser desorption/ionization time of flight mass spectrometry profile dendrogram of selected pyogenic and zoonotic streptococci from study of human infections caused by unusual strains of *Streptococcus halichoeri*, United States. We compared main spectra profiles from *S. halichoeri* isolates to Bruker Biotyper (https://www.bruker.com) database entries from related streptococcal species. Distance level indicates relative similarity of mass spectral profiles. ^T^ indicates type strains.

We generated MALDI-TOF MSPs from the 5 selected *S. halichoeri* isolates and compared them with Bruker Biotyper database entries from related streptococcal species. The MSP dendrogram (Figure 2) shows that the *S. halichoeri* clinical isolates are readily distinguishable from other streptococci and cluster closely together with the *S. halichoeri* subsp. *halichoeri*–type strain. This suggests that the proposed subspecies are not currently distinguishable by MALDI-TOF mass spectrometry.

## Discussion

The genus *Streptococcus* is composed of >130 species of gram-positive, catalase-negative bacteria that are found in a variety of environments. Several streptococcal species cause zoonotic infections in humans, including *S. canis*, *Streptococcus equi* subsp. *zooepidemicus*, *S. iniae*, and *S. suis* (*10*–*13*). *S. halichoeri* is one of the more recently identified zoonotic streptococci, first described in 2004 after isolation from gray seals (*H. grypus*) (*1*). It was originally described as a gram-positive, catalase-negative, nonhemolytic organism, occurring in pairs or short chains, and expressing Lancefield group B antigen; this combination did not correspond to any previously identified *Streptococcus* species. 16S rRNA gene sequencing confirmed its placement in the genus *Streptococcus*, but the >3% divergence from described taxa suggested it was a novel species (*1*). *S. halichoeri* was also isolated from a European badger with pyogranulomatous pleuropneumonia (*9*) and from several canine and fur-producing animal species, including companion animals (*14*,*15*). These observations indicate a much broader host range than originally described for this organism.

The clinical laboratories at the Marshfield Clinic in Wisconsin have isolated this organism from both canine (n = 5) and feline (n = 1) clinical specimens (*16*). All isolates were nonhemolytic, had streptococcal morphology on Gram stain, were catalase and PYR-positive, and typed as Lancefield group B. Although initially reported as “*Streptococcus* species, unable to identify further,” they were subsequently identified as *S. halichoeri* by MALDI-TOF mass spectrometry and 16S rRNA gene sequencing.

The first reported human infection with *S. halichoeri* was in a man with diabetes who sought treatment for an empyema; he reported handling fish before his illness (*2*). *S*. *halichoeri* was isolated from pleural fluid cultures as tiny, white, nonhemolytic colonies of gram-positive cocci in chains that could not be identified by standard phenotypic methods or 16S rRNA sequencing. The organism was positive for Lancefield group B antigen and was ultimately identified as *S. halichoeri* by MALDI-TOF mass spectrometry. The isolate was sensitive to penicillin and levofloxacin, and the patient’s condition was successfully treated with 4 weeks of intravenous ceftriaxone after 10 days of levofloxacin.

Another reported human case was of an 84-year-old man with a history of diabetes who sought treatment for bacterial cellulitis of the left thigh following prior treatment for left-sided endocarditis and cellulitis at the same site due to *S*. *agalactiae* (*17*). Blood and wound culture isolates were initially identified as *S. pyogenes* by VITEK 2 and *S. agalactiae* by API 20 STREP (bioMérieux). The discordance in identification necessitated analysis at a reference laboratory where the isolates were identified as *S. halichoeri* by MALDI-TOF mass spectrometry but as *S. suis* by VITEK 2. Ultimately, both were confirmed as *S. halichoeri* by *sodA* and 16S rRNA gene sequencing. The isolates were sensitive to penicillin, levofloxacin, and linezolid but resistant to erythromycin, clindamycin, and tetracycline. The patient’s condition was successfully treated with 15 days of amoxicillin.

Finally, a 2016 publication with limited clinical information highlighted 6 additional isolates recovered from human specimens (*3*). Most (4/6) isolates were from blood and were shown to have homogenous phenotypes, sequence at multiple loci, and genomic similarities consistent with a difference at the subspecies level from the originally described *S. halichoeri* isolate, leading to proposal of the new taxon, *S. halichoeri* subsp. *hominis*. AST data on the 6 human isolates revealed susceptibility to most drugs tested, although 1 isolate was erythromycin resistant and 2 were tetracycline resistant.

It is unclear if *S. halichoeri* represents an emerging zoonosis or a rare but still underdiagnosed infection, or if it is simply being recognized only now because of improved identification methods (e.g., MALDI-TOF mass spectrometry). The source for infection with *S. halichoeri* is certainly not limited to marine life, because the organism has been isolated from both domestic and wild terrestrial mammals (*14*). Patients in the cases presented here had no known exposures to animals; therefore, reservoirs for infection by *S. halichoeri* might be more widespread than once thought.

*S. halichoeri* was originally described as catalase-negative, but our testing confirmed reports that it can be catalase-positive when grown on blood-supplemented media. This observation is not unprecedented for *Streptococcus*-like organisms; some streptococci and enterococci can be weakly catalase-positive when grown on media containing blood (*4*). However, some *S. halichoeri* isolates are strongly catalase-positive under these conditions, leading to the risk of misidentification (Table 1). A 2020 study identified genes in some *S. halichoeri* isolates highly similar to known catalase genes, potentially explaining these phenotypic observations (*14*). Whereas phenotypic methods may be misleading, MALDI-TOF mass spectrometry is a rapid and reliable method for identifying *S. halichoeri* (Figure 2). As the MALDI-TOF mass spectrometry method becomes more widely available, we may begin to identify more infections caused by *S. halichoeri* or other underrecognized bacteria (*6*). In the absence of MALDI-TOF mass spectrometry, laboratories should consider further testing on atypical isolates from deep wound and sterile body sites when they are phenotypically identified as coagulase-negative staphylococci but morphologically consistent with streptococci.

*S. halichoeri* subsp. *hominis* isolates were described as positive for bile-esculin, esculin, and acid from sucrose fermentation, but *S. halichoeri* subsp. *halichoeri* was negative for all 3 tests (*3*), suggesting possible phenotypic distinction of the subspecies. The 5 clinical isolates we characterized were positive for bile-esculin and esculin; 4 of them (isolates 018, 116, 229, and 583) were most closely related to *S. halichoeri* subsp. *hominis* by full-length 16S sequencing. Surprisingly, isolate 076 was positive for bile-esculin and esculin, like *S. halichoeri* subsp. *hominis*, but was nearly identical to the full-length 16S rRNA sequences from *S. halichoeri* subsp. *halichoeri* from both gray seals and badgers. This apparent discrepancy suggests that isolate 076 was a phenotypic variant of the *S. halichoeri* subsp. *halichoeri*. However, because of limited published data, the reliability of esculin or other phenotypic tests for distinguishing these proposed subspecies is unknown. Unfortunately, we do not have information on recent zoonotic exposures for isolate 076.

There are several parallels between the patients reported in our study, 2 of whom had diabetes, and the patients from the 2 previously published cases, both of whom also had diabetes (*2*,*17*). These similarities may indicate an opportunistic nature of this organism, in which establishing infection requires diabetes or an immunocompromised state. A similar association with diabetes mellitus has been observed among fishmongers who have contracted zoonotic infections through occupational exposures (*13*). Diabetes mellitus is also one of the most common coexisting health conditions associated with group B *Streptococcus* infection (*18*,*19*). One patient from this study and 1 from another study (*2*) had purulent infections, which may indicate pathogenic potential similar to the pyogenic streptococci, to which *S. halichoeri* is closely related (*20*). 

All isolates in this study, as well as those in previous reports, were susceptible to β-lactams and levofloxacin, which may be considered antimicrobials of choice; daptomycin, linezolid, and vancomycin also showed very good antimicrobial activity. Our AST analysis showed that 5 (23%) of 22 isolates were resistant to erythromycin and 4 (18%) were nonsusceptible to doxycycline (when CLSI breakpoints for *S. pneumoniae* were applied) (*5*), similar to observations made elsewhere (*3*). This analysis highlights the relative antimicrobial susceptibility of *S. halichoeri* isolates, but the exceptions point out the need to monitor the susceptibility patterns of this emerging pathogen. Given its phenotypic similarities with coagulase-negative staphylococci and the viridans group streptococci, it is likely that *S. halichoeri* continues to be disregarded by some as a skin commensal rather than a true cause of infection. In addition, our study highlights the importance of recognizing *S. halichoeri* infections and the role of MALDI-TOF mass spectrometry and 16S rRNA gene sequencing in accurately identifying this pathogen.
